# Primary diffuse large B-cell lymphoma of orbit: A population-based analysis

**DOI:** 10.3389/fmed.2022.990538

**Published:** 2022-09-15

**Authors:** Yu-Qing Chen, Zi-Fan Yue, Sai-Nan Chen, Fei Tong, Wei-Hua Yang, Rui-Li Wei

**Affiliations:** ^1^Department of Ophthalmology, Changzheng Hospital of Naval Medicine University, Shanghai, China; ^2^Shenzhen Eye Hospital, Jinan University, Shenzhen, China; ^3^Eye Hospital, Nanjing Medical University, Nanjing, China

**Keywords:** diffuse large B-cell lymphoma, primary orbital lymphoma, survival, prognosis, SEER

## Abstract

**Objective:**

Primary orbital lymphoma (POL) accounts for an essential part of adult orbital malignancies. Nevertheless, it remains a relatively rare lymphoid malignancy, accounting for <1% of all non-Hodgkin's lymphoma (NHL) cases. Orbital diffuse large B-cell lymphoma (DLBCL) is one of the most prevalent subtypes of POL that confers the worst outcomes. The prognostic determinants of orbital DLBCL remain unknown. Therefore, a retrospective analysis was conducted by investigating the Surveillance, Epidemiology, and End Results (SEER) database for independent predictive factors for the prognosis of orbital DLBCL.

**Materials and methods:**

Using the SEER program, we acquired patient data including demographics, clinical characteristics, and treatment strategies. Our cohort included cases of primary orbital DLBCL diagnosed from 2000 to 2017. We conducted Kaplan-Meier analyses to visualize the overall survival (OS) and cause-specific survival (CSS). The Cox proportional hazard regression models were applied to assess the effects of these prognostic factors on OS and CSS.

**Results:**

The present cohort included 332 patients with orbital DLBCL. Age was the most impacted variable by orbital DLBCL. Three independent prognostic variables of orbital DLBCL were identified on diagnosis: advanced age, no radiation treatment, and late-stage (Stage IV). Moreover, patients who underwent chemotherapy demonstrated a greater OS when compared with those who did not. In orbital DLBCL, being unmarried was also a poor prognostic factor.

**Conclusion:**

The current study is the largest population-based case series of orbital DLBCL. The age at the time of diagnosis, marital status, absence of chemotherapy or radiotherapy, and tumor stage were all found to be correlated with worse prognosis.

## Introduction

Primary orbital lymphoma (POL) is a common and the most prevalent orbital malignancy affecting older adults (1). Nevertheless, among all primary sites of the extra-nodal lymphoma, the orbit remains a relatively rare one, accounting for <1% of all non-Hodgkin's lymphoma (NHL) cases (2). Diffuse large B-cell lymphoma (DLBCL) is one of the most common histological subtypes of POL associated with the worst outcomes (3). However, the prognostic determinants of orbital DLBCL remain to be fully elucidated.

Since the orbital DLBCL is uncommon, efforts to clarify its clinical characteristics and the prognostic outcomes mostly rely on case reports and the collections of patients' cohorts from across the world (4–10). The clinical presentation, treatment strategies, and survival in orbital DLBCL have not been adequately analyzed at a large population level. Consequently, we sought to identify prognostic factors and the overall survival (OS) trends in orbital DLBCL by searching a national registry database—The SEER database. The National Cancer Institute developed the SEER program, which collects valuable information on cancer incidence and survival outcomes in USA, encompassing ~28% of all USA residents (11, 12). As of May 30, 2022, this research is the largest population-based case series of orbital DLBCL aimed at evaluating the clinical features and OS characteristics as well as determining the correlated prognostic factors in orbital DLBCL.

## Methods

### Data source and cohort selection

We adopted the 3rd edition of the International Classification of Diseases for Oncology [ICD-O-3] histology codes (9680/3, 9684/3, 9688/3, 9735/3, 9737/3, and 9738/3) to identify cases of orbital DLBCL and then accessed the interesting cohort. The original sites of DLBCL were determined by clinical physicians. We identified the primary orbital DLBCL by searching site number C69.6 (orbit, not otherwise specified [NOS]). We carried out this study following the standard guidelines and under the approval of the Ethics Committee of Shanghai Changzheng Hospital, Second Military Medical University.

All enrolled cases were diagnosed *via* valid histological identification, and cases that were diagnosed solely based on clinical characteristics, radiographic examination, autopsy, or death certificate were excluded. In addition, we excluded patients whose survival time was no more than 1 month. A resembling screen procedure was then conducted to acquire the comparative cohort of nodal DLBCL and extra-nodal DLBCL (without orbit).

The relevant data on survival, outcome, chemotherapy, radiotherapy, surgeries, the 6th edition of stage group-derived American Joint Committee on Cancer (AJCC), the laterality of orbit involvement, sex, race, age of diagnosis, and marital status were also queried. AJCC-6 staging was conducted instead of the updated AJCC-7 staging and AJCC-8 staging, as the former contains more data on DLBCL patients. Data on grading was sparse or unavailable. Surgical inventions were grouped as follows: surgery (codes: 10-80, 90, 98), no surgery (codes: 00), or unknown (codes: 99). Chemotherapy, as well as radiotherapy, were separately grouped as “yes” or “no/unknown”.

### Outcomes and statistical analyses

The demographic characteristics and treatments of patients in the cohort were computed using descriptive statistics, and the differences among extra-nodal DLBCL, nodal DLBCL, and orbital DLBCL were analyzed by ANOVA, Chi-squared test, and Kruskal-Wallis test. We evaluated the survival outcomes, including OS and CSS, through Kaplan-Meier survival models and log-rank test. We determined the prognostic factors by using the univariate and multivariate Cox proportional hazard regression models. Hazard ratio (HR) with 95% confidence intervals (95% CI) were applied to explore the prognostic factors on OS and CSS. RStudio (RStudio, Inc., Boston, MA) was conducted for statistical analyses, and the *p* < 0.05 was considered significant.

## Results

### Incidence in primary orbital DLBCL

From 2000 to 2017, we collected 2024 cases of primary orbital NHL, including 332 (16.40%) cases of orbital DLBCL. Mucosal-Associated Lymphoid Tissue (MALT) Lymphoma (*n* = 1,048, 51.78%), and Follicular Lymphoma (*n* = 237, 11.71%) were the other top 3 histological subtypes. From 2000 to 2017, 332 cases of orbital DLBCL accounted for 0.34% of primary DLBCL (*n* = 97,607) and 1.00% of primary extra-nodal DLBCL (*n* = 33,060).

### Clinical features and treatment

We enrolled 332 patients with primary orbital DLBCL meeting the inclusion criteria in our study. Table 1 summarizes the clinical and demographical features of the orbital, nodal, and other extra-nodal (non-orbit) DLBCL. The average age (68.9 years) of orbital DLBCL patients was greater than that of nodular DLBCL patients (64.1 years) and other extra-nodal DLBCL patients (65.4 years). There were equivalent cases of women (50.0%) diagnosed with orbital DLBCL (*n* = 166, sex-ratio: 1.00). However, DLBCL was more likely to affect men (about 55%) than the other two types of DLBCLs. The majority of the orbital DLBCL patients (82.8%) were Caucasians, which was consistent with the result for extra-nodal and nodal DLBCL patients. More than half of the patients in the entire cohort, as well as in the other two equivalents, were married. According to the Derived AJCC Stage Group, 6th Edition, the majority of the patients were classified into the following four stages: stage I (43.1%), stage IV(17.5%), stage II (4.2%), and stage III (1.8%). Chemotherapy was the primary treatment administered to patients with orbital DLBCL (66.0%), but it was also the least common among these 3 types of malignancies. Radiotherapy was the preferred treatment for 159 (47.9%) patients with orbital DBLCL, which is much higher than that for nodal DLBCL (17.9%) and other extra-nodal DLBCL (27.8%). Finally, only 131 (39.5%) patients received the surgical interventions in orbital DLBCL.

**Table 1:** Demographics and clinical features of patients with orbital DLBCL, nodal, and other extra-nodal sites as diagnosed in the SEER database (2000–2017).

	**Orbital DLBCL (*N =* 332)**	**Extranodal DLBCL (*N =* 32728)**	**Nodal DLBCL (*N =* 64547)**	***P*-value**
**Age**
Mean (SD)	68.9(15.6)	65.4 (16.5)	64.1 (16.4)	<0.001***
Median [Min, Max]	70.0 [15.0, 97.0]	68.0 [0, 100]	66.0 [0, 100]	
**Age**
<60	84 (25.3%)	10,404 (31.8%)	22,593 (35.0%)	<0.001***
60–75	118 (35.5%)	12,133 (37.1%)	24,186(37.5%)	
>75	130 (39.2%)	10,191 (31.1%)	17,768 (27.5%)	
**Gender**
Male	166 (50.0%)	17897 (54.7%)	35493 (55.0%)	0.135
Female	166 (50.0%)	14831 (45.3%)	29054 (45.0%)	
**Race**
White	275 (82.8%)	27,290 (83.4%)	54,377 (84.2%)	<0.001***
Black	27 (8.1%)	2,122 (6.5%)	4,929 (7.6%)	
API	28 (8.4%)	2,969 (9.1%)	4,579 (7.1%)	
AI/AN	0 (0%)	176 (0.5%)	346 (0.5%)	
Unknown	2 (0.6%)	171 (0.5%)	316 (0.5%)	
**Marital status**
Married	172 (51.8%)	17,925 (54.8%)	35,797 (55.5%)	0.0017**
Single	48 (14.5%)	5,275 (16.1%)	10,589 (16.4%)	
Unmarried	88 (26.5%)	7,684 (23.5%)	14,857 (23.0%)	
Unknown	24 (7.2%)	1,844 (5.6%)	3,304 (5.1%)	
**Stage group**
I	143 (43.1%)	10,699 (32.7%)	7,371 (11.4%)	<0.001***
II	14 (4.2%)	4,349 (13.3%)	8,773 (13.6%)	
III	6 (1.8%)	1,119 (3.4%)	10,187 (15.8%)	
IV	58 (17.5%)	5,380 (16.4%)	15,548 (24.1%)	
Unknown	111 (33.4%)	11,181 (34.2%)	22,668 (35.1%)	
**Surgery**
Yes	131 (39.5%)	11,986 (36.6%)	16,692 (25.9%)	<0.001***
No	198 (59.6%)	20,575 (62.9%)	47,590 (73.7%)	
Unknown	3 (0.9%)	167 (0.5%)	265 (0.4%)	
**Chemotherapy**
Yes	219 (66.0%)	24,513 (74.9%)	53,121 (82.3%)	<0.001***
No/Unknown	113 (34.0%)	8,215 (25.1%)	11,426 (17.7%)	
**Radiation**
Yes	159 (47.9%)	9,094 (27.8%)	11,551 (17.9%)	<0.001***
No/Unknown	173 (52.1%)	23,634 (72.2%)	52,996 (82.1%)	

DLBCL, diffuse large B cell lymphoma; API, Asian or Pacific Islander; AI/AN, American Indian/Alaska Native; NA, not available.

*p < 0.05, **p < 0.01, ***p < 0.001, * indicate that demographic and clinical characteristics were not identical in orbital DLBCL, nodal, and other extra-nodal DLBCL.

### Survival

**Figure 1:**
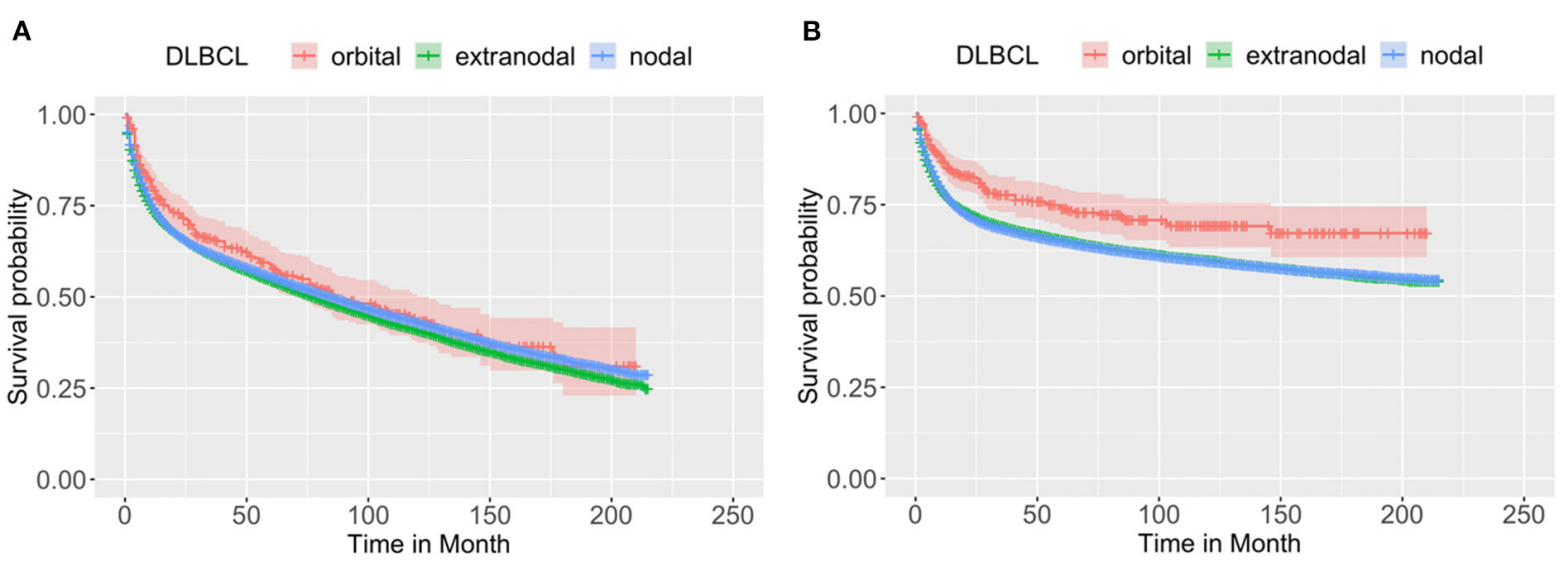
The Kaplan–Meier survival curve of **(A)** OS and **(B)** CSS of patients with DLBCL by the primary sites: orbit (*n* = 332), other extra-nodal sites (*n* = 32,728), and nodal site (*n* = 64,547). Through log-rank test, it was revealed that the OS and CSS were not identical among the three groups, with a *p* < 0.0001 and 0.0012, respectively, indicating the primary sites may affect the survival of DLBCL patients.

Figure 1 shows the comparison of the OS (Figure 1A) and CSS (Figure 1B) of the orbital DLBCL patients to nodal/other extra-nodal DLBCL patients, demonstrating that the primary sites may affect the survival of DLBCL patients. All cases with orbital DLBCL in our cohort had a 3-, 5-, and 10-year OS of 65.99, 57.85, and 43.29%, respectively. Table 2 depicts statistical data on the OS probability of orbital DLBCL, extra-nodal DLBCL (without orbit), and nodal DLBCL. When compared to the other two types of DLBCL, patients with orbital DLBCL demonstrated a superior survival rate. Not only did orbital DLBCL confer the greatest OS but also provided the greatest CSS after 3-, 5-, and 10- years (77.67, 73.86, and 69.14%, respectively). Figure 1 displays the Kaplan–Meier curves of OS and CSS among these 3 DLBCLs.

**Table 2:** Univariate and multivariate analysis of factors related to the risks of OS for patients with DLBCL in the SEER Program database from 2000 to 2017.

**Variables**	**Univariate analysis**		**Multivariate analysis**	
	**HR (95% CI)**	***P*-value**	**HR (95% CI)**	***P*-value**
Age range			
<60	Reference		Reference	
60–75	1.979(1.932–2.028)	<0.001***	2.007(1.957–2.058)	<0.001***
>75	4.115(4.017–4.215)	<0.001***	3.909(3.808–4.012)	<0.001***
**Gender**				
Male	Reference		Reference	
Female	0.976(0.959–0.993)	0.007**	0.814(0.798–0.829)	<0.001***
**Marital status**				
Married	Reference		Reference	
Single	0.893(0.869–0.917)	<0.001***	1.193(1.160–1.228)	<0.001***
Unmarried	1.614(1.581–1.647)	<0.001***	1.307(1.278–1.336)	<0.001***
Unknown	1.051(1.008–1.095)	0.019*	0.937(0.898–0.978)	0.003**
**Race**				
White	Reference		Reference	
Black	0.973(0.940–1.007)	0.119	1.246(1.202–1.291)	<0.001***
API	0.954(0.922–0.988)	0.008**	1.023(0.989–1.059)	0.191
AI/AN	1.112(0.989–1.251)	0.077	1.304(1.158–1.470)	<0.001***
Unknown	0.198(0.152–0.257)	<0.001***	0.207(0.159–0.271)	<0.001***
**DLBCL types**
Orbital DLBCL	Reference		Reference	
Extranodal DLBCL	1.148(0.985–1.339)	0.077	1.213(1.039–1.416)	0.015*
**(Without Orbital DLBCL)**				
Nodal DLBCL	1.088(0.933–1.268)	0.281	1.115(0.954–1.302)	0.172

OS, overall survival; DLBCL, diffuse large B cell lymphoma; SEER, Surveillance, Epidemiology, and End Results; HR, hazard ratio; CI, confidence interval; API, Asian or Pacific Islander; AI/AN, American Indian/Alaska Native; NA, not available.

*p < 0.05, **p < 0.01, ***p < 0.001, * indicate that demographic and clinical characteristics were not identical in orbital DLBCL, nodal, and other extra-nodal DLBCL.

### Univariate/multivariate analysis for predicting independent prognostic factors

**Figure 2:**
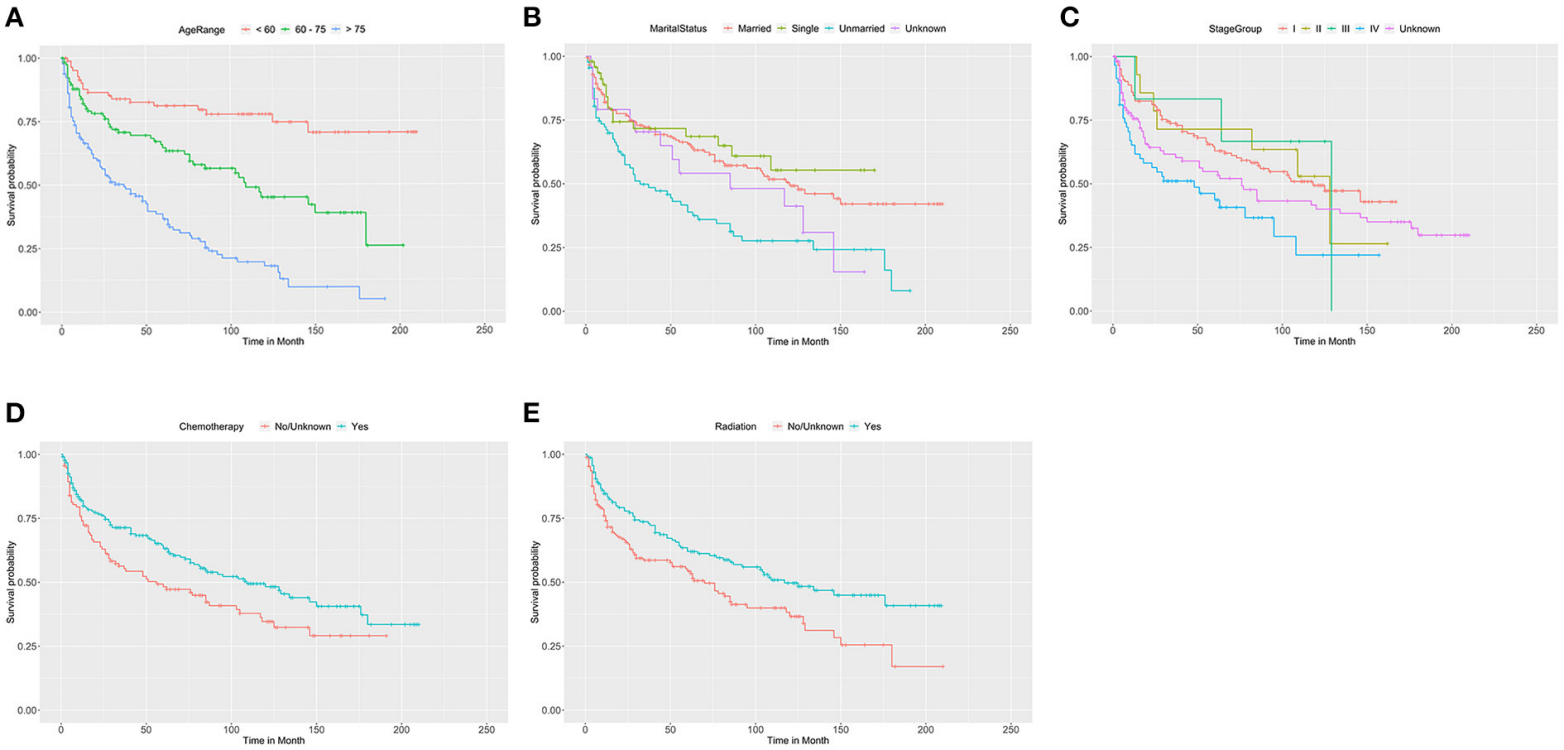
The Kaplan-Meier survival curve of OS in patients with orbital DLBCL stratifed by **(A)** age at diagnosis, **(B)** marital status, **(C)** stage, **(D)** chemotherapy, and **(E)** radiation. It was revealed that older age, unmarried status and stage IV were associated with worse OS compared by log-rank test. In **(A)**, 60-75 vs<60 years (*p* < 0.001); >75 vs. < 60 years (*p* < 0.001). In **(B)**, Unmarried vs. Married (*p* < 0.001); Single vs. Married (*p* = 0.520); Unknown vs. Married (*p* = 0.221). In **(C)**, stage IV vs. stage I (*p* = 0.001); stage II vs. stage I (*p* = 0.919); and stage III vs. stage I (*p* = 0.853), and unknown vs. stage I (*p* = 0.089). In **(D,E)**, chemotherapy (*p* = 0.016) and radiation therapy (*p* = 0.004) had significantly better OS than those without it.

**Figure 3:**
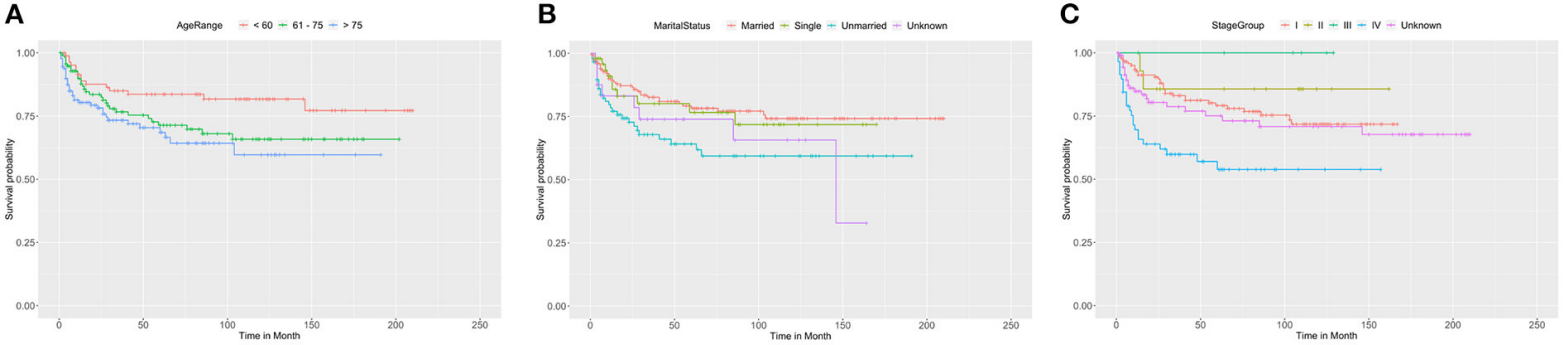
The Kaplan-Meier survival curve of CSS in patients with orbital DLBCL stratifed by **(A)** age at diagnosis, **(B)** marital status, **(C)** stage. It was revealed that old age, unmarried status, and stage IV were associated with worse CSS compared by log-rank test. In **(A)**, >75 vs. <60 years (*p* = 0.007); 60-75 vs< 60 years (*p* = 0.074). In **(B)**, Unmarried vs. Married (*p* = 0.006); Single vs. Married (*p* = 0.787); Unknown vs. Married (*p* = 0.161). In **(C)**, stage IV vs. stage I (*p* < 0.001); stage II vs. stage I (*p* = 0.460); stage III vs. stage I (*p* = 0.994); unknown vs. stage I (*p* = 0.345).

We adopted univariate survival analysis to demonstrate increased age at diagnosis as a prognostic factor for OS (60–75 vs. <60 years, HR = 2.628, 95% CI 1.549–4.458, *p* < 0.001; >75 vs. <60 years, HR = 6.026, 95% CI 3.654–9.937, *p* < 0.001) (Figure 2A). Moreover, the Cox proportional hazard regressions indicated that patients age ≥75 years served as a crucial prognostic factor for CSS (HR = 2.324, 95% CI 1.265–4.270, *p* = 0.007) (Figure 3A). Furthermore, we noted remarkable differences in OS and CSS between married and unmarried (widowed/divorced) groups of orbital DLBCL patients (OS: unmarried vs. married, HR = 1.997, 95% CI 1.420–2.808, *p* < 0.001; CSS: unmarried vs. married, HR = 2.002, 95% CI 1.218–3.290, *p* = 0.006) (Figures 2B, 3B). When we compared OS by the AJCC stages in orbital DLBCL, we found that stage IV orbital DLBCL was related to a higher risk of death [OS: stage IV vs. stage I, HR = 2.022, 95% CI 1.336–3.062, *p* < 0.001 (Figure 2C); CSS: stage IV vs. stage I, HR = 2.612, 95% CI 1.529–4.462, *p* < 0.001 (Figure 3C)] (Tables 3, 4). In terms of treatment strategies, chemotherapy was also a prognostic factor for higher OS (HR = 0.683, 95% CI 0.501–0.932, *p* = 0.016) (Figure 2D), but not CSS (HR = 0.796, 95% CI 0.508–1.245 *p* = 0.317) (Table 4); similarly, patients with radiotherapy demonstrated greater OS (HR = 0.636, 95% CI 0.466–0.866, *p* = 0.004) (Figure 2E) than those without it, but not CSS (HR = 0.737, 95% CI 0.475–1.143, *p* = 0.173) (Table 4). However, we found little evidence supporting that surgical interventions improved the survival outcomes. The gender and race of the patients, as well as the laterality of the malignancy, were not linked with mortality (Tables 3, 4).

**Table 3:** Univariate and multivariate analysis of factors with the risk of OS for orbital DLBCL patients identified in the SEER Program database from 2000 to 2017.

**Variables**	**Univariate analysis**		**Multivariate analysis**	
	**HR (95% CI)**	***P*-value**	**HR (95% CI)**	***P*-value**
**Age range**
**<60**	Reference		Reference	
**60–75**	2.628(1.549–4.458)	<0.001***	2.508(1.458–4.318)	<0.001***
**>75**	6.026(3.654–9.937)	<0.001***	4.888(2.815–8.489)	<0.001***
**Gender**
**Male**	Reference			
**Female**	1.238(0.911–1.682)	0.172		
**Laterality**
**Bilateral**	Reference			
**Single**	0.610(0.286–1.302)	0.201		
**Others**	1.140(0.235–5.521)	0.871		
**Marital status**
**Married**	Reference		Reference	
**Single**	0.837(0.488–1.438)	0.52	1.082(0.620–1.890)	0.781
**Unmarried**	1.997(1.420–2.808)	<0.001***	1.260(0.859–1.848)	0.237
**Unknown**	1.429(0.807–2.53)	0.221	1.140(0.629–2.066)	0.665
**Race**
**White**	Reference			
**Black**	0.820(0.444–1.517)	0.528		
**API**	1.395(0.831–2.343)	0.208		
**AI/AN**	NA	NA		
**Unknown**	NA	0.995		
**Stage Group**
**I**	Reference			
**II**	0.960(0.440–2.096)	0.919	0.827(0.348–1.963)	0.667
**III**	0.896(0.281–2.855)	0.853	0.625(0.193–2.022)	0.433
**IV**	2.022(1.336–3.062)	0.001***	1.747(1.130–2.702)	0.012*
**Unknown**	1.374(0.953–1.980)	0.089	1.180(0.814–1.709)	0.382
**Chemotherapy**
**No/Unknown**	Reference		Reference	
**Yes**	0.683(0.501–0.932)	0.016*	0.727(0.522–1.012)	0.059
**Radiation No/Unknown**
**Yes**	Reference			
**Surgery**	0.636(0.466–0.866)	0.004**	0.666(0.477–0.928)	0.016*
**No**
**Unknown**	Reference			
**Yes**	1.706(0.420–6.940)	0.455		
	0.921(0.673–1.260)	0.608		

OS, overall survival; DLBCL, diffuse large B cell lymphoma; SEER, Surveillance, Epidemiology, and End Results; HR, hazard ratio; CI, confidence interval; API, Asian or Pacific Islander; AI/AN, American Indian/Alaska Native; NA, not available.

*p < 0.05, **p < 0.01, ***p < 0.001.

**Table 4:** Univariate and multivariate analysis of factors with the risk of CSS for orbital DLBCL patients identified in the SEER Program database from 2000 to 2017.

**Variables**	**Univariate analysis**		**Multivariate analysis**	
	**HR (95% CI)**	***P*-value**	**HR (95% CI)**	***P*-value**
**Age Range**
<60	Reference		Reference	
60–75	1.761(0.946–3.277)	0.074	1.752(0.923–3.326)	0.086
>75	2.324(1.265–4.270)	0.007**	1.742(0.870–3.487)	0.117
**Gender**
Male	Reference			
Female	1.215(0.785–1.882)	0.382		
**Laterality**
Bilateral	Reference			
Single	0.430(0.174–1.064)	0.068		
Others	1.981(0.382–10.274)	0.416		
**Marital status**
Married	Reference		Reference	
Single	1.102(0.544–2.232)	0.787	1.250(0.603–2.593)	0.548
Unmarried	2.002(1.218–3.290)	0.006**	1.776(1.018–3.099)	0.043*
Unknown	1.735(0.803–3.749)	0.161	1.856(0.850–4.054)	0.121
**Race**
White	Reference			
Black	0.747(0.301–1.855)	0.53		
API	1.529(0.762–3.069)	0.232		
AI/AN	NA	NA		
Unknown	NA	NA		
**Stage Group**
I	Reference			
II	0.583(0.140–2.439)	0.46	0.323(0.044–2.382)	0.267
III	0(0-lnf)	0.994	0 (0-lnf)	0.994
IV	2.612(1.529–4.462)	<0.001***	2.575(1.485–4.465)	0.001***
Unknown	1.294(0.757–2.212)	0.345	1.140(0.664–1.957)	0.634
**Chemotherapy**
No/Unknown	Reference			
Yes	0.796(0.508–1.245)	0.317		
**Radiation**
No/Unknown	Reference			
Yes	0.737(0.475–1.143)	0.173		
**Surgery**
No	Reference			
Yes	0.816(0.516–1.289)	0.383		
Unknown	2.845(0.690–11.720)	0.148		

CSS, cancer-specific survival; DLBCL, diffuse large B cell lymphoma; SEER, Surveillance, Epidemiology, and End Results; HR, hazard ratio; CI, confidence interval; API, Asian or Pacific Islander; AI/AN, American Indian/Alaska Native; NA, not available.

*p < 0.05, **p < 0.01, ***p < 0.001.

Tables 3, 4 display the results of multivariate survival analysis in summary. The advanced age of diagnosis (dichotomized at <60, 61–75, and >75 years) acted as an independent prognostic factor of OS (61–75 vs. <60 years, HR = 2.508, 95% CI 1.458–4.318, *p* = 0.001; >75 vs. <60 years, HR = 4.888, 95% CI 2.815–8.489, *p* < 0.001). Furthermore, stage IV orbital DLBCL patients were more likely to experience worse survival than stage I patients; we found that in both OS (HR = 1.747, 95% CI 1.130–2.702, *p* = 0.012) (Table 3) and CSS (HR = 2.575, 95% CI 1.485–4.465, *p* = 0.001) (Table 4). Radiotherapy was an independent prognostic factor indicating greater OS (HR = 0.666, 95% CI 0.477–0.928, *p* = 0.016) (Table 3). In addition, widowed or divorced patients showed lower CSS (HR = 1.776, 95% CI 1.018–3.099, *p* = 0.043) than married patients (Table 4).

## Discussion

DLBCL is acknowledged as a malignancy of mature B lymphocytes and is one of the most prevalent subtypes of NHL, accounting for 25% of all NHL cases (13). DLBCL has been shown to be more frequent among male patients (56.0%) and Whites (85.5%) in the US (14). The predominant primary site of DLBCL was nodal in ~58% of the cases, extra-nodal in 42%, and extra-nodal extramedullary in 40% (15, 16). Orbital DLBCL is the second-most common NHL in orbit and a high-grade aggressive malignancy with the worst prognosis of any POL subtype (10). Several primary sites of extra-nodal DLBCL involvement, including the lung, kidney, adrenal, and ovary, are frequently aggressive and indicate disseminated diseases. Thyroid, gastric, and orbital DLBCL, on the other hand, indicate a relatively better prognosis under standard treatments (17). Our study is the first to investigate the clinical characteristics and prognosis of orbital DLBCL in such a large cohort.

It was found that the mean age of orbital DLBCL patients (68.9 years) exceeded the mean age of both nodal DLBCL (64.1 years) and other extra-nodal DLBCL patients (65.4 years) in this study. Thus, it is reasonable to suspect orbital DLBCL in these elderly patients. These results revealed that patients aged more than 60 years were more tend to have orbital DLBCL. In addition, the age range of our cohort patients was 15 to 97years (median age: 70 years), which is older than the median age of nodal DLBCL and extra-nodal DLBCL (66.0 and 68.0 years, respectively). We found that patients with extra-nodal DLBCL were remarkably older than those nodal DLBCL patients (15). Accordingly, we speculated that patients with orbital DLBCL represent one of the subgroups of extra-nodal DLBCL that arises later in life. Several factors might have contributed to this situation. First, the rate of inflammatory progression may have increased over time, which is currently recognized as a prominent risk for morbidity and mortality in the elderly (18). Moreover, it has been demonstrated that older age was an independent predictor of worse CSS in primary testicular DLBCL (19). It has been proven, particularly, that older age is an important predictor for patients with orbital lymphomas (3). Past studies have shown that advanced age conferred a poor prognosis in orbital lymphomas. The 5-year OS rate in this research was 57.85%, which is consistent with the findings in the former DLBCL research, wherein ocular adnexal DLBCL patients received the same treatments and showed a 5-year OS rate of 54–55.9% (20, 21). The outcomes of the univariate analysis revealed that patients above the age of 60 years had significantly worse OS and CSS. Furthermore, the result of multivariate analysis showed that older age was an independent predictor for worse OS in orbital DLBCL patients, but not for worse CSS.

Moreover, we observed that unmarried status was an independent predictor for poor outcomes in orbital DLBCL patients, which supported the theory that unmarried, widowed, or divorced patients had a greater risk of disease-related death and tumor metastases than married patients (22). Widowed Hodgkin lymphoma (HL) patients had poorer survival outcomes than married or others, and their marital status indicated an essential role in HL prognosis (23). Psychosocial factors might explain why marital status is relevant with a worse prognosis in lymphoma patients. Stress has been demonstrated to influence physical health (24, 25). Insufficient psychosocial support and excessive psychological stress were discovered to jeopardize the immune functions and resulted in death (26, 27). As psychosocial stress may be one of the predominant factors contributing to the high mortality rate of widowed or divorced patients, greater social support and assistance are needed for these patients.

Notably, the tumor stage can affect the survival outcome of orbital DLBCL. In both univariate and multivariate analyses, the advanced stage (stage IV) was related to a worse prognosis in our current investigation. Patients with stage IV orbital DLBCL showed lower OS and CSS than those with stage I lymphoma (20).

Orbital DLBCL, like other types of lymphoma, is treated with chemotherapy, radiotherapy, and surgery (1, 28). Chemotherapy is the most commonly utilized treatment in this cohort and notably improved prognosis. These findings were consistent with the findings of other extra-nodal DLBCL types, such as primary renal DLBCL (29), urinary tract DLBCL (30), adrenal DLBCL (31), and intestinal DLBCL (32). Furthermore, chemotherapy is the predominantly used treatment option for nodal DLBCL (33). Nevertheless, a former study demonstrated that the effectiveness of chemotherapy was determined by the primary site of extra-nodal NHL; they found no significant improvement among genitourinary DLBCL patients who received chemotherapy treatment (34). However, the combination of surgery and chemotherapy may cause a variance, and there is no specific data illustrating the application of other treatments in this case. Therefore, we need to conduct more valid studies in the future to elucidate the benefit of chemotherapy on the survival of patients with orbital DLBCL.

Furthermore, radiation was found to be advantageous for the survival of orbital DLBCL patients in our investigation. The patients who received radiation manifested more significant outcomes than those who did not. More than half of the orbital lymphoma patients with high-grade DLBCL (56.0%) received radiotherapy compared to 47.9% in our cohort (1). Nevertheless, our data lacked specific information on the frequency and extent of the radiation. Thus, additional studies are warranted to determine the influence of radiation treatment on the prognosis of orbital DLBCL.

We also observed that surgery did not influence the survival outcomes of patients with orbital DLBCL. Surgery is majorly performed in conjunction with other treatment modalities, particularly chemotherapy or radiotherapy. Surgery is not always employed in the treatment of orbital lymphoma and seldom used as the only treatment modality. It was reported that 30% of DLBCLs receive an excision (total or partial) of the neoplasm (1). The data we collected from the SEER database did not describe the specific surgical types, and it was difficult to clarify the survival disparities noted in the current cohort. Since orbitotomy surgery includes biopsy, the majority of the procedures in our cohort were most likely merely biopsies that did not affect the prognosis.

There were several limitations in our study. First, it was a retrospective analysis, the retrospective data has intrinsic reporting biases. Second, chemotherapy is the mainstay of treatment for the management of orbital DLBCL. However, records on chemotherapy delivery are sparse, and recovery of these missing data may potentially upgrade our study's prediction model. There were also main limitations of the SEER chemotherapy and radiotherapy data, such as the completeness of the variables, and the biases associated with who receives treatment. The other limitation of the study is the unavailability of data about the laboratory values, clinical course, and the International Prognostic Index (IPI) (35). Furthermore, the disease's natural history was not complete in all cases (36). It, therefore, remains ambiguous whether the difference in our cohort's survival outcomes was influenced by these factors or by other unaccounted confounding factors. Thus, cohort studies with a larger sample size are warranted to validate the present findings.

## Conclusion

In summary, the present study is the largest cohort to clarify the influences of demographic characteristics and clinical presentations on the prognosis of patients with orbital DLBCL. We hope that, through comparison of orbital DLBCL to nodal DLBCL and extra-nodal DLBCL (without the orbit) patients, we may assist clinical ophthalmologists and oncologists in better managing patients with orbital DLBCL. Older age, unmarried status, and stage IV were identified as the crucial factors conferring a poor prognosis of primary orbital DLBCL. Patients treated with chemotherapy or radiation therapy showed a longer life expectancy than those who did not. Further studies are however needed to validate the present observations.

## Data availability statement

The original contributions presented in the study are included in the article/supplementary material, further enquiries can be directed to the corresponding author/s.

## Ethics statement

Written informed consent was obtained from the individual(s) for the publication of any potentially identifiable images or data included in this article.

## Author contributions

Y-QC, FT, and R-LW contributed to the conception, design, and drafted the manuscript. Y-QC, Z-FY, and S-NC analyzed the data. Y-QC, W-HY, and R-LW contributed with a critical revision of the manuscript. All authors contributed to the article and approved the submitted version.

## Funding

This study was funded by grants from the National Natural Science Foundation of China (81770959 and 81570885), Shenzhen Fund for Guangdong Provincial High-level Clinical Key Specialties (SZGSP014), and Sanming Project of Medicine in Shenzhen (SZSM202011015).

## Conflict of interest

The authors declare that the research was conducted in the absence of any commercial or financial relationships that could be construed as a potential conflict of interest.

## Publisher's note

All claims expressed in this article are solely those of the authors and do not necessarily represent those of their affiliated organizations, or those of the publisher, the editors and the reviewers. Any product that may be evaluated in this article, or claim that may be made by its manufacturer, is not guaranteed or endorsed by the publisher.
